# miR-27a and miR-27b regulate autophagic clearance of damaged mitochondria by targeting PTEN-induced putative kinase 1 (PINK1)

**DOI:** 10.1186/s13024-016-0121-4

**Published:** 2016-07-26

**Authors:** Jaekwang Kim, Fabienne C. Fiesel, Krystal C. Belmonte, Roman Hudec, Wang-Xia Wang, Chaeyoung Kim, Peter T. Nelson, Wolfdieter Springer, Jungsu Kim

**Affiliations:** 1 Department of Neuroscience, Mayo Clinic College of Medicine, 4500 San Pablo Rd S, Jacksonville, FL 32224 USA; 2 Department of Pathology, University of Kentucky, Lexington, KY 40536 USA; 3 Neurobiology of Disease Program, Mayo Graduate School, Jacksonville, FL 32224 USA

**Keywords:** PINK1, Mitophagy, miR-27a, miR-27b, Parkinson’s disease

## Abstract

**Background:**

Loss-of-function mutations in *PINK1* and *PARKIN* are the most common causes of autosomal recessive Parkinson’s disease (PD). PINK1 is a mitochondrial serine/threonine kinase that plays a critical role in mitophagy, a selective autophagic clearance of damaged mitochondria. Accumulating evidence suggests mitochondrial dysfunction is one of central mechanisms underlying PD pathogenesis. Therefore, identifying regulatory mechanisms of PINK1 expression may provide novel therapeutic opportunities for PD. Although post-translational stabilization of PINK1 upon mitochondrial damage has been extensively studied, little is known about the regulation mechanism of PINK1 at the transcriptional or translational levels.

**Results:**

Here, we demonstrated that microRNA-27a (miR-27a) and miR-27b suppress PINK1 expression at the translational level through directly binding to the 3′-untranslated region (3′UTR) of its mRNA. Importantly, our data demonstrated that translation of PINK1 is critical for its accumulation upon mitochondrial damage. The accumulation of PINK1 upon mitochondrial damage was strongly regulated by expression levels of miR-27a and miR-27b. miR-27a and miR-27b prevent mitophagic influx by suppressing PINK1 expression, as evidenced by the decrease of ubiquitin phosphorylation, Parkin translocation, and LC3-II accumulation in damaged mitochondria. Consequently, miR-27a and miR-27b inhibit lysosomal degradation of the damaged mitochondria, as shown by the decrease of the delivery of damaged mitochondria to lysosome and the degradation of cytochrome c oxidase 2 (COX2), a mitochondrial marker. Furthermore, our data demonstrated that the expression of miR-27a and miR-27b is significantly induced under chronic mitophagic flux, suggesting a negative feedback regulation between PINK1-mediated mitophagy and miR-27a and miR-27b.

**Conclusions:**

We demonstrated that miR-27a and miR-27b regulate PINK1 expression and autophagic clearance of damaged mitochondria. Our data further support a novel negative regulatory mechanism of PINK1-mediated mitophagy by miR-27a and miR-27b. Therefore, our results considerably advance our understanding of PINK1 expression and mitophagy regulation and suggest that miR-27a and miR-27b may represent potential therapeutic targets for PD.

**Electronic supplementary material:**

The online version of this article (doi:10.1186/s13024-016-0121-4) contains supplementary material, which is available to authorized users.

## Background

Parkinson’s disease (PD), the second most common neurodegenerative disorder, is clinically manifested by motor symptoms, such as bradykinesia, resting tremor, rigidity, and postural instability [1]. PD is pathologically characterized by the loss of dopaminergic neurons in substantia nigra *pars compacta* (SN) [1]. Identification of several genetic risk factors has led to a significant advance in our understanding of PD pathogenesis. To date, mutations in several genes, such as *SNCA (α-synuclein), Leucine-rich repeat kinase 2 (LRRK2), DJ-1, PARK2 (PARKIN),* and PTEN-induced putative kinase 1 (*PINK1*) [1], have been shown to cause familial early-onset PD, accounting for 5–10 % of all cases [2].

Mounting evidence suggests mitochondrial dysfunction as a central mechanism in PD pathogenesis [3]. For instance, mitochondrial toxins, such as MPTP and rotenone, induce parkinsonism in animal models and human [4]. Moreover, damage to mitochondrial DNA in SN is increased in aging and PD patients [5, 6]. Furthermore, the activity of complex I is reduced in SN of PD patients [7, 8]. Therefore, a better understanding of how the integrity and function of mitochondria are maintained following insults may provide novel opportunities for PD treatment.

Mutations in *PARKIN* and *PINK1* are the leading causes of autosomal recessive PD [9, 10]. Together, they functionally mediate the stress-activated, selective clearance of impaired mitochondria via autophagic degradation (termed mitophagy) [11], supporting the idea that deficits in mitochondria quality control may be a core mechanism underlying PD pathogenesis. PINK1, a mitochondrial serine/threonine kinase, is maintained at low levels under normal physiological conditions. Upon mitochondrial damage, PINK1 is quickly stabilized on damaged mitochondria, which leads to translocation of cytosolic Parkin to damaged mitochondria [11–13]. PINK1 activates Parkin by phosphorylating it at Ser65. Once activated, Parkin, an E3 ubiquitin ligase, selectively tags the damaged mitochondria for mitophagic clearance by ubiquitinating multiple mitochondrial proteins [14, 15]. Recently, several groups discovered that PINK1 also phosphorylates ubiquitin at Ser65 (pUb^S65^) and PINK1-dependent phosphorylation of ubiquitin is required for translocation and activation of Parkin to initiate mitophagy [16–18]. Moreover, pUb^S65^ has been suggested to accumulate with mitochondrial stress, age, and disease [19] and to serve as a specific signal for recognition by autophagy receptors [20, 21]. Therefore, accumulation of pUb^S65^ is a hallmark of mitochondrial stress/damage. Beyond their roles in mitophagy, mounting evidence suggested that both PINK1 and Parkin have neuroprotective roles against various insults, such as oxidative stress and PD-relevant toxins, although the underlying mechanisms still remain elusive [22, 23].

Given its critical roles in mitochondrial homeostasis and neuroprotection, the mechanism of how *PINK1* expression is regulated has been under intense investigations. Although PINK1 regulation at the post-translational level has been extensively studied [24], little is known about the regulation mechanism of PINK1 at transcriptional or translational levels. microRNAs (miRNAs) have been gaining growing attention as key regulators of protein coding genes [25, 26]. Small non-coding miRNAs, ~22 nucleotides, are generated from primary transcripts via sequential cleavages by Drosha, ribonuclease type III (DROSHA) and Dicer 1, ribonuclease type III (DICER1) complexes [27]. miRNAs regulate expression of their target genes by binding to cognate messenger RNAs (mRNAs), leading to translational repression [27]. Thus far, a few miRNAs are identified as regulators of PD-associated genes, such as *SNCA*, *LRRK2*, and *DJ-1* [28–31]. However, post-transcriptional regulation of human PINK1 by miRNAs is poorly defined.

Here, we identified a novel regulatory mechanism of PINK1 by miR-27a/b via targeting its 3′UTR. We demonstrated that miR-27a and miR-27b inhibit PINK1 stabilization, thereby preventing autophagic degradation of impaired mitochondria.

## Results

### PINK1 expression is regulated by miRNAs

**Fig. 1:**
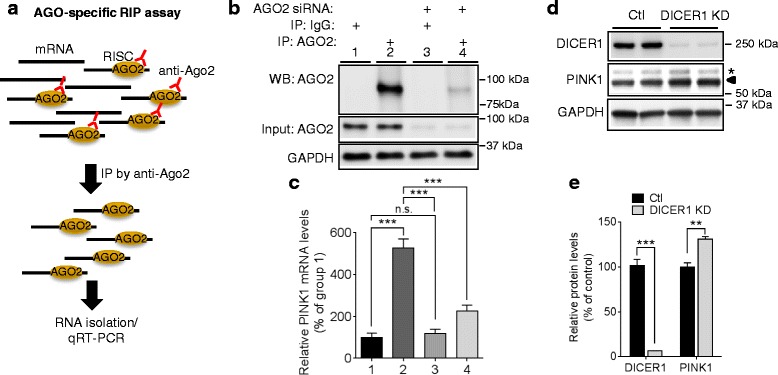
Regulation of PINK1 by miRNA. **a** Schematic diagram of AGO-specific RNA immunoprecipitation assay. **b**, **c** *PINK1* mRNAs are associated with miRNA/RISC complex. HeLa cells were transfected with 50 nM of AGO2 siRNA or negative control. 48 h post-transfection, AGO2-bound mRNAs were pulled down from cell lysates with 2A8 anti-AGO2 antibody or non-specific IgG antibody. *PINK1* mRNA levels were then measured by qRT-PCR. siRNA against AGO2 served as a quality control for AGO-specific RNA immunoprecipitation assay. *PINK1* mRNA levels were quantified as a percentage of group 1 (*n* = 6, two-way ANOVA). **d**, **e** Knock-down of *DICER1* increased PINK1 protein levels. HeLa cells were transfected with 50 nM *DICER1* siRNA (DICER1 KD) or negative control (Ctl). 48 h post-transfection, cells were harvested for Western blot. The arrowhead indicates a full-length PINK1 band and the asterisk indicates a non-specific band. Each protein level was normalized to corresponding GAPDH level and quantified as a percentage of control (*n* = 4, *t*-test). Values are mean ± SEM (n.s. = non-significant, **p < 0.01, ***p < 0.001)

*PINK1* mRNA (NM_032409) has a long 3′UTR, ~840 nucleotides, raising the possibility that its expression may be regulated by miRNAs which often target 3′UTR sequences. The mature miRNAs bind to their target mRNAs through incorporation into the RNA-induced silencing complex (RISC) which is composed of Argonaute (AGO) and several other partners [32]. To determine if PINK1 expression is regulated by miRNAs, we first assessed whether human *PINK1* mRNA is associated with RISC complex using AGO-specific RNA immunoprecipitation method in HeLa cells (Fig. 1a). AGO2-bound miRNA/mRNA complexes were first pulled down with an AGO-specific antibody. Non-specific IgG served as negative control. *PINK1* mRNA levels were then analyzed by qRT-PCR. Pull-down with anti-AGO antibody resulted in dramatic increase of *PINK1* mRNA levels compared to the control IgG, whereas *AGO2* knock-down with siRNA markedly decreased *PINK1* mRNA levels pulled down by anti-AGO antibody (Fig. 1b, c). We next tested whether inhibition of miRNA biogenesis via *DICER1* knock-down would affect PINK1 protein levels. As expected, *DICER1* knock-down decreased miRNA levels in HeLa cells (Additional file 1). Compared to the negative control, *DICER1* knock-down with siRNA significantly increased PINK1 protein levels in HeLa cells (Fig. 1d, e). Collectively, our data indicate that human PINK1 expression is regulated by miRNAs.

### miR-27a/b suppress human PINK1 expression by directly targeting 3′UTR of its mRNA

**Fig. 2:**
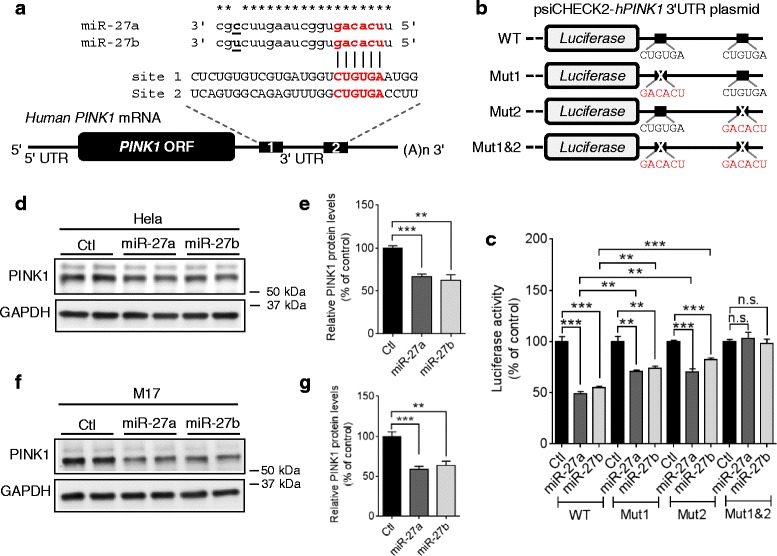
miR-27a/b suppress human PINK1 expression. **a** Schematic diagram of the conserved target sites of miR-27a/b in the 3′UTR of human *PINK1* mRNA. Seed sequences were indicated in red. Asterisks (*) indicate the conserved nucleotides. **b** Schematic diagram of the luciferase reporter plasmids. The reporter constructs contain the full-length 3′UTR of human *PINK1* mRNA with wild-type (WT) or mutated (Mut) seed match sites downstream of *Renilla* luciferase gene. **c** Overexpression of miR-27a/b decreased luciferase activities in HeLa cells. Cells were transfected with miR-27a/b or negative control (Ctl) along with reporter constructs as indicated. 48 h post-transfection, luciferase activities were measured (*n* = 4, one-way ANOVA). *Renilla* luciferase activity was normalized to the corresponding *firefly* luciferase activity. **d**-**g** Overexpression of miR-27a/b decreased PINK1 protein levels in HeLa (D-E) and M17 cells (F-G). Cells were transfected with miR-27a/b or negative control (Ctl). 48 h post-transfection, cells were harvested for Western blot (*n* = 4, *t*-test). PINK1 protein level was normalized to corresponding GAPDH level and quantified as a percentage of control. Data are shown as a percentage of control. Values are mean ± SEM (n.s. = non-significant, **p < 0.01, ***p < 0.001)

To identify potential miRNAs that directly regulate PINK1 expression, we first searched miRNAs that have putative binding sites in the 3′UTR of human *PINK1* mRNA by utilizing several miRNA-target prediction algorithms as described previously [33]. Among several candidates, we focused on miR-27a and miR-27b (hereafter referred to as miR-27a/b) because 1) miR-27a/b are commonly predicted by several algorithms to have seed match sequences in the 3′UTR of human *PINK1* mRNA (Fig. 2a and Additional file 2a), 2) miR-27a/b are expressed in SN of human midbrain (Additional file 2a) [34–36], 3) miR-27a/b have multiple putative binding sites in the 3′UTR of human *PINK1* mRNA with low binding free energies (Additional file 2b) [37]. Of note, both putative binding sites of miR-27a/b in the 3′UTR of *PINK1* mRNA are well conserved in primates, such as human, chimpanzee, and monkey, but not in rodents, such as rat and mouse (Additional file 3), raising the possibility of primate-specific regulation of PINK1 expression by miR-27a/b.

To determine if miR-27a/b directly suppress PINK1 expression by targeting its 3′UTR, we performed a luciferase assay with the reporter construct containing the entire 3′UTR of human *PINK1* mRNA downstream of luciferase (Fig. 2b). miR-27a/b significantly suppressed the expression of luciferase in HeLa cells (Fig. 2c). Mutations in either seed match site1 or site2 significantly increased luciferase activities compared to wild-type (Fig. 2c). Moreover, a combined mutation in both seed match sites (Mut 1&2) completely abolished the miR-27a/b-mediated reduction of luciferase activities, indicating that both seed match sites are functional (Fig. 2c). There was no difference in the levels of miR-27a and miR-27b expressed between the miR transfected groups (Additional file 4). To determine if miR-27a/b affect PINK1 protein levels, we first transfected synthetic miR-27a, miR-27b, or negative control to human HeLa cells (Additional file 5a). Overexpression of miR-27a/b markedly decreased PINK1 protein levels in HeLa cells (Fig. 2d, e). A combination of miR-27a and miR-27b did not further decrease PINK1 levels in HeLa cells, suggesting that both miR-27a and miR-27b target the same site in the 3′UTR of *PINK1* mRNA (Additional file 6). To evaluate the effect of miR-27a/b in another cell type, we tested M17 cells. miR-27a/b also suppressed PINK1 expression in human dopaminergic-like M17 cells (Fig. 2f, g).

**Fig. 3:**
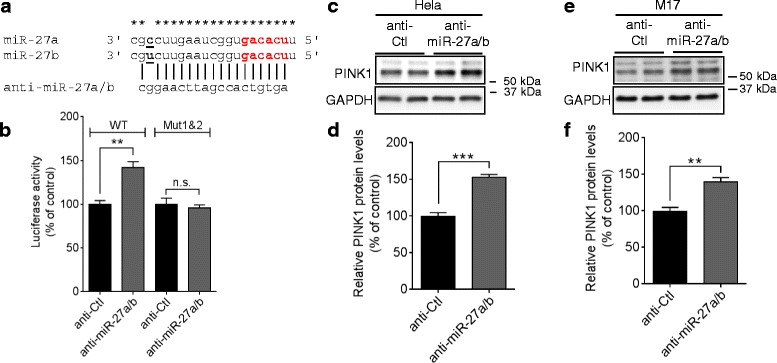
Inhibition of endogenous miR-27a/b function increases human PINK1 expression. **a** Schematic diagram of sequence alignment of miR-27a/b and anti-miR-27a/b. Asterisks (*) indicate conserved nucleotides. **b** Inhibition of endogenous miR-27a/b function increased luciferase activities in HeLa cells. Cells were transfected with the reporter constructs containing the full-length 3′UTR of human *PINK1* mRNA with wild-type (WT) or mutated (Mut) seed match sites downstream of *Renilla* luciferase gene. 48 h post-transfection, luciferase activities were measured (*n* = 4, *t*-test). **c**-**f** Inhibition of endogenous miR-27a/b increased PINK1 protein levels in HeLa (C-D) and M17 cells (E-F). Cells were transfected with 150 nM of anti-miR-27a/b or anti-control (anti-Ctl). 48 h post-transfection, cells were harvested for Western blot. PINK1 protein level was normalized to corresponding GAPDH level and quantified as a percentage of control (*n* = 4, *t*-test). Data are shown as a percentage of control. Values are mean ± SEM (n.s. = non-significant, **p < 0.01, ***p < 0.00)

Next, we examined whether endogenous miR-27a/b regulate PINK1 expression. To inhibit miR-27a/b function, we used locked nucleic acid (LNA)-based anti-miR that is nuclease-resistant single-stranded antisense oligonucleotides complementary to their target miRNAs [38]. Due to the high similarity in the mature sequences of miR-27a and miR-27b, single anti-miR (denoted as anti-miR-27a/b) inhibited both miR-27a and miR-27b (Fig. 3a and Additional file 5b). Inhibition of endogenous miR-27a/b significantly increased luciferase activities from the reporter construct with WT *PINK1* 3′UTR, whereas combined mutations of both seed match sites blocked the anti-miR-27a/b-mediated induction of luciferase activities in HeLa cells (Fig. 3b). Moreover, Inhibition of endogenous miR-27a/b significantly increased PINK1 protein levels in HeLa cells as well as M17 cells (Fig. 3c-f). Collectively, these data indicate that miR-27a/b suppress human PINK1 expression by directly targeting 3′UTR of its mRNA.

### PINK1 translation is indispensable for the accumulation of PINK1 upon mitochondrial damage

**Fig. 4:**
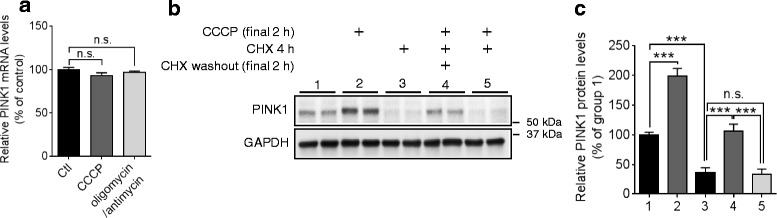
PINK1 translation is critical for the accumulation of PINK1 upon mitochondrial damage. **a** *PINK1* mRNA levels were not altered by mitochondrial damage. HeLa cells were incubated with 10 μM CCCP or combination of 10 μM oligomycin and 4 μM antimycin for 2 h and then, *PINK1* mRNA levels were measured by qRT-PCR. *PINK1* mRNA level was normalized to the corresponding *GAPDH* levels and quantified as a percentage of control (*n* = 8, *t*-test). **b**, **c** Translation is involved in PINK1 accumulation upon CCCP treatment. HeLa cells were first pre-incubated with 100 μM cycloheximide (CHX) for 2 h and then washed. Cells were further incubated with 10 μM CCCP with or without CHX for 2 h (*n* = 4, two-way ANOVA). Values are mean ± SEM (n.s. = non-significant, ***p < 0.001)

PINK1 protein is rapidly and constitutively degraded in healthy mitochondria by mitochondrial proteases, whereas mitochondrial damage allows PINK1 to accumulate on the outer membrane of the damaged mitochondria [39]. However, it has not been well determined whether transcription and/or translation step of *PINK1* may contribute to the accumulation of PINK1 protein upon mitochondrial damage. Given a low basal level of PINK1 protein and a rapid build-up of PINK1 protein upon mitochondrial damage, we hypothesized that new synthesis of PINK1 mRNA together with post-translational stabilization may contribute to the accumulation of PINK1 upon mitochondrial damage. Thus, we first examined whether PINK1 mRNA levels are altered by mitochondrial damage. PINK1 mRNA levels were not affected by mitochondrial damages induced by either carbonyl cyanide m-chlorophenylhydrazone (CCCP, mitochondrial uncoupler) or combination of oligomycin (inhibitor of ATP synthase) and antimycin (inhibitor of mitochondrial respiratory chain complex III) in HeLa cells (Fig. 4a). Notably, blocking translation with cycloheximide (CHX) completely abolished the accumulation of PINK1 protein by CCCP (Fig. 4b, c). Taken together, these data suggest that PINK1 translation is critical for the accumulation of PINK1 upon mitochondrial damage.

### miR-27a/b inhibit PINK1 accumulation upon mitochondrial damage

**Fig. 5:**
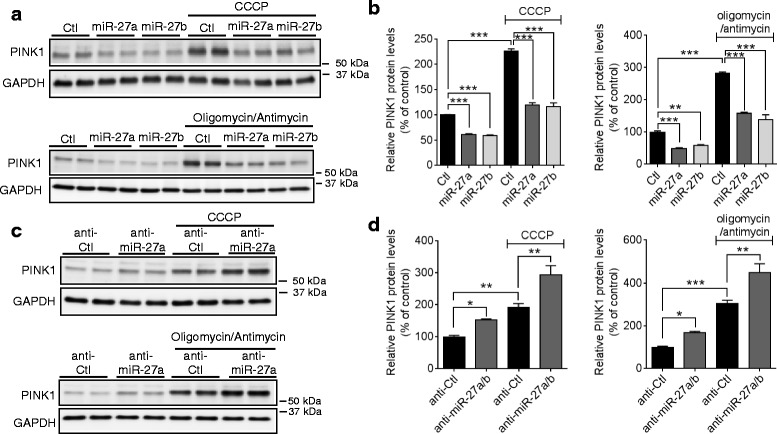
miR-27a/b inhibit PINK1 accumulation upon mitochondrial damage. **a**, **b** Overexpression of miR-27a/b inhibited PINK1 accumulation upon mitochondrial damage. 48 h post-transfection, HeLa cells were treated with 10 μM CCCP or combination of 10 μM oligomycin and 4 μM antimycin as indicated for 2 h (*n* = 4, two-way ANOVA). **c**, **d** Inhibition of endogenous miR-27a/b increased PINK1 accumulation upon CCCP treatment. (*n* = 4, two-way ANOVA). PINK1 levels were normalized to corresponding GAPDH level and quantified as a percentage of control. Values are mean ± SEM (n.s. = non-significant, *p < 0.05, **p < 0.01, ***p < 0.001)

Because miR-27a/b suppress PINK1 expression at the translational level, we next examined whether miR-27a/b influence PINK1 accumulation upon mitochondrial damage. miR-27a/b-mediated suppression of PINK1 dramatically reduced the PINK1 accumulation upon CCCP treatment compared to the negative control (Fig. 5a, b), whereas inhibition of miR-27a/b further increased PINK1 accumulation compared to the negative control (Fig. 5c, d). Furthermore, miR-27a/b showed similar effects on PINK1 accumulation upon combined treatment of oligomycin and antimycin (Fig. 5a-d). Taken together, our data suggest that the accumulation of PINK1 upon mitochondrial damage is strongly regulated by expression levels of miR-27a and miR-27b.

### miR-27a/b prevent the accumulation of phospho-ubiquitin upon mitochondrial damage

**Fig. 6:**
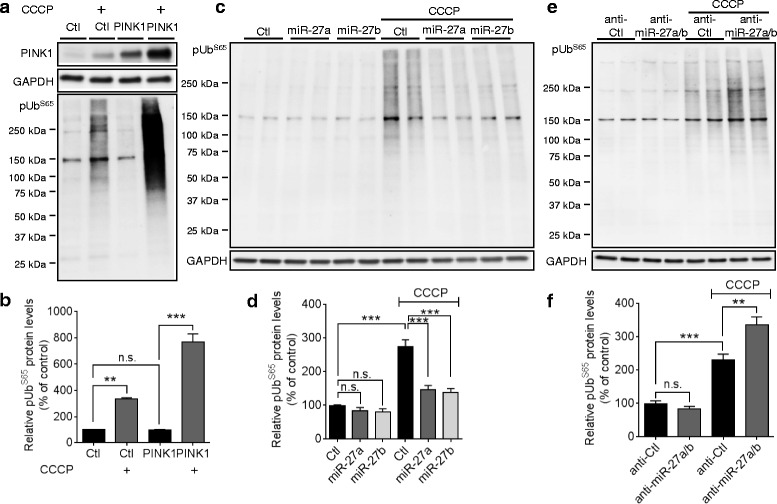
miR-27a/b prevent pUb^S65^ accumulation upon mitochondrial damage. **a**, **b** Overexpression of PINK1 increased pUb^S65^ accumulation in HeLa cells only when incubated with CCCP. 48 h post-transfection, cells were incubated with 10 μM CCCP for 2 h and pUb^S65^ levels were determined by Western blot (*n* = 3, two-way ANOVA). **c**, **d** Overexpression of miR-27a/b inhibited pUb^S65^ accumulation by CCCP in HeLa cells (*n* = 4, two-way ANOVA). **e**, **f** Inhibition of endogenous miR-27a/b increased pUb^S65^ accumulation by CCCP in HeLa cells (*n* = 4, two-way ANOVA). PINK1 levels were normalized to corresponding GAPDH level and quantified as a percentage of control. Values are mean ± SEM (n.s. = non-significant, **p < 0.01, ***p < 0.001)

Recent studies demonstrated that PINK1 phosphorylates ubiquitin at Ser65 (pUb^S65^) upon mitochondrial damage [16–18]. Ubiquitin phosphorylation by PINK1 plays a critical role in the activation and mitochondrial translocation of Parkin [16–18]. We also observed that pUb^S65^ levels were markedly increased by CCCP and overexpression of PINK1 further increased the pUb^S65^ accumulation by CCCP in HeLa cells (Fig. 6a). However, overexpression of PINK1 did not affect pUb^S65^ levels under the basal condition without CCCP treatment (Fig. 6a), suggesting that ubiquitin kinase activity of PINK1 is activated specifically upon mitochondrial damage.

In line with these data, overexpression of miR-27a/b inhibited the accumulation of pUb^S65^ upon CCCP treatment compared to the negative control (Fig. 6c, d), whereas inhibition of miR-27a/b further increased pUb^S65^ levels in the presence of CCCP compared to the negative control (Fig. 6e, f). In contrast, pUb^S65^ levels were not affected by either overexpression or inhibition of miR-27a/b under the basal condition without CCCP treatment (Fig. 6c-f). Taken together, our data demonstrate that miR-27a/b prevent the accumulation of pUb^S65^ upon mitochondrial damage by suppressing *PINK1* expression.

### miR-27a/b prevent Parkin translocation to mitochondria upon mitochondrial damage

**Fig. 7:**
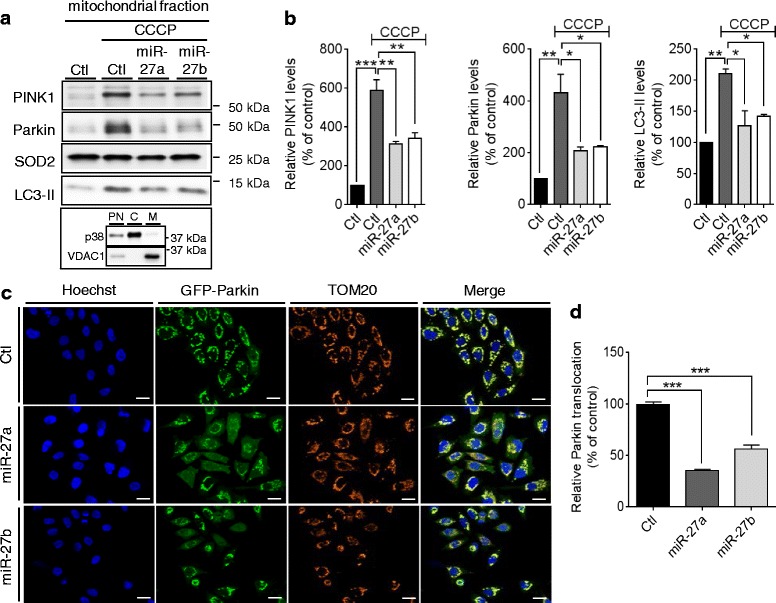
miR-27a/b prevent Parkin translocation to mitochondria upon mitochondrial damage. **a**, **b** 48 h post-transfection, HeLa cells were incubated with 10 μM CCCP for 2 h. After isolating the mitochondria-enriched fractions, Parkin translocation and LC3-II were assessed by Western blot. Each protein level was normalized to corresponding mitochondrial loading control (SOD2) level and quantified as a percentage of control (*n* = 3, two-way ANOVA). Purity of mitochondrial fraction was assessed by monitoring p38 cytosolic and VDAC1 mitochondrial markers. PN; post-nuclear, C; cytoplasm, M; mitochondrial (A). **c**, **d** 48 h post-transfection, HeLa cells stably expressing GFP-Parkin were visualized by GFP, a mitochondrial marker (TOM20) antibody, and a nuclear dye (Hoechst) as indicated. Scale bars correspond to 10 μm. Parkin translocation was quantified as the ratio of cytoplasmic to nuclear GFP signal and the resulting ratios were normalized to control. Data were collected from 4 independent replicates (n > 1000 cells) and are shown as a percentage of control. Values are mean ± SEM (*p < 0.05, **p < 0.01, ***p < 0.001)

PINK1-dependent translocation of cytosolic Parkin to damaged mitochondria is a prerequisite for mitophagy [11]. To determine if miR-27a/b prevent Parkin translocation upon mitochondrial damage, we transfected miR-27a/b or negative control to HeLa cells stably expressing Parkin. After inducing mitochondrial damage by CCCP for 2 h, mitochondrial fractions were separated from total cell lysates and then analyzed by Western blot (Fig. 7a). Compared to the negative control, miR-27a/b strongly inhibited Parkin translocation to mitochondria as well as accumulation of active form of LC3 (LC3-II), an autophagic marker, upon CCCP treatment (Fig. 7a, b).

To confirm our biochemical analysis of miR-27a/b effect on Parkin translocation, we also quantified Parkin translocation by High Content Imaging. After transfected with a negative control or miR-27a/b, HeLa cells expressing GFP-Parkin were treated with CCCP for 2 h, stained with a nuclear dye, and imaged. Parkin translocation was measured by assessing the ratio of cytoplasmic and nuclear GFP signal as described previously [40]. miR-27a/b overexpression markedly decreased the levels of Parkin colocalized with mitochondria marker TOM20, compared to the negative control (Fig. 7c, d). Taken together, our data demonstrate that miR-27a/b prevent the induction of mitophagy by suppressing PINK1 expression.

### miR-27a/b inhibit the lysosomal degradation of damaged mitochondria

**Fig. 8:**
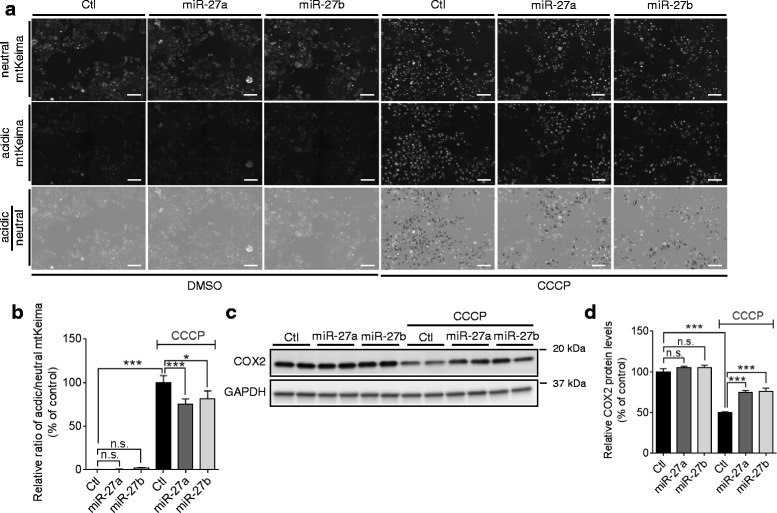
miR-27a/b inhibit the degradation of mitochondria via lysosome upon mitochondrial damage. **a**, **b** miR-27a/b inhibit the delivery of damaged mitochondria to lysosome. 48 h post-transfection, HeLa cells stably expressing mtKeima were treated with CCCP or DMSO for 12 h. Cells were sequentially scanned using 440/10 nm (neutral) and 548/20 nm (acidic) excitation filters. Scale bars correspond to 100 μm. The signal intensity of the acidic Keima was divided by the intensity of the neutral mtKeima. Data were collected from 12 independent replicates and are shown as a percentage of control (n > 300 cells, two-way ANOVA). **c**, **d** miR-27a/b inhibit the degradation of COX2 upon mitochondrial damage. 48 h post-transfection, HeLa cells were treated with 10 μM CCCP or DMSO for 16 h. The degradation of damaged mitochondria was assessed by monitoring the levels of COX2, a mitochondrial inner membrane protein, by Western blot (*n* = 4, two-way ANOVA). Values are mean ± SEM (n.s. = non-significant, *p < 0.05, ***p < 0.001)

Damaged mitochondria are selectively tagged by a combination of PINK1 and Parkin and then, delivered to lysosome for hydrolytic degradation [11]. Therefore, we next examined whether miR-27a/b inhibit the delivery of damaged mitochondria to lysosome by using mitochondria-targeting Keima (mtKeima) [41]. Keima is a fluorescent protein derived from coral which is resistant to lysosomal proteases and changes its color upon pH change. mtKeima is engineered to localize in mitochondria and changes its color upon a fusion of neutral autophagosome with acidic lysosome [41]. After transfected with a negative control, PINK1 siRNA, or miRNAs as indicated, HeLa cells expressing mtKeima were treated with CCCP for 12 h and imaged. Lysosomal delivery of damaged mitochondria was measured by assessing the ratio of acidic and neural Keima signal as described previously [41]. As a positive control for the assay, we first tested the effect of PINK1 knockdown. PINK1 knockdown dramatically decreased the lysosomal delivery of damaged mitochondria, validating our assay performance (Additional file 7a, b). miR-27a/b overexpression significantly decreased the lysosomal delivery of damaged mitochondria (Fig. 8a, b). Autophagic clearance of damaged mitochondria was further assessed by monitoring the degradation of COX2, a mitochondrial inner membrane protein. miR-27a/b inhibited the degradation of COX2 upon mitochondrial damage, compared to a negative control miR (Fig. 8c, d). These two independent assays indicate that miR-27a/b inhibit autophagic degradation of damaged mitochondria by lysosome.

### Induction of miR-27a/b under the chronic mitophagic flux condition

**Fig. 9:**
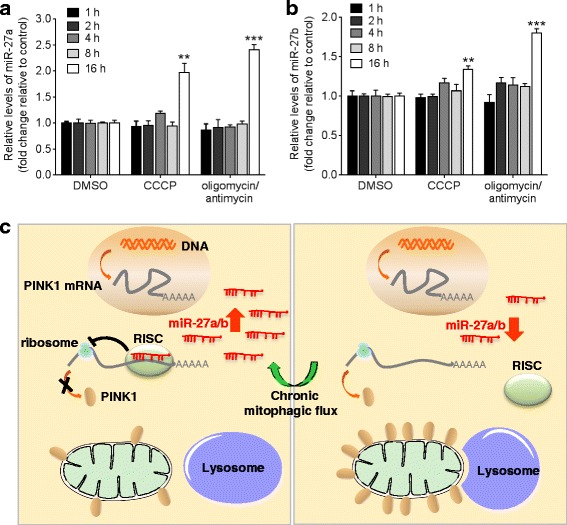
The levels of miR-27a/b are increased under chronic mitophagic flux condition. **a**, **b** HeLa cells were incubated with DMSO, 10 μM CCCP, or combination of 10 μM oligomycin and 4 μM antimycin as indicated. miR-27a/b levels were measured by qRT-PCR and normalized to corresponding *U6* levels. Data are shown as fold changes relative to the DMSO controls. Values are mean ± SEM (*t*-test, **p < 0.01, ***p < 0.001). **c** A proposed role of miR-27a/b in mitophagy. Upon chronic mitophagic flux, the levels of miR-27a/b are increased and miR-27a/b function as a negative feedback loop to turn off excessive mitophagic flux. Upon the induction of miR-27a/b expression, the translation of PINK1 mRNA is inhibited by miR-27a/b. Consequently, the level of PINK1 protein in mitochondria decreases and thereby, autophagic degradation of the damaged mitochondria by lysosome is inhibited (*Left panel*). In contrast, PINK1 translation and lysosomal clearance of damaged mitochondria are increased when PINK1 inhibition mediated by miR-27a/b was disinhibited by the low levels of miR-27a/b (*Right panel*)

To determine if there is a regulatory interaction between miR-27a/b and mitophagy, we next examined the change in miR-27a/b levels upon mitochondrial damage at different time points. Interestingly, the levels of miR-27a/b were dramatically increased under the chronic mitophagic flux condition in HeLa cells (Fig. 9a, b). miR-27a and miR-27b are produced from miR-23a ~ 27a ~ 24–2 cluster at chromosome 19p13 and miR-23b ~ 27b ~ 24–1 cluster at chromosome 9q22, respectively (Additional file 8a). The mature miRNAs of miR-23a ~ 27a ~ 24–2 cluster are known to be derived from a single primary transcript [42]. The miRNAs of miR-23b ~ 27b ~ 24–1 cluster are transcribed independently but regulated by the same promoter [43]. Therefore, the expression of miRNAs in these clusters is expected to be regulated together. In the same manner with miR-27a/b, the levels of miR-23a/b were also increased under the chronic mitophagic flux condition (Additional file 8b, c). However, there was no change in the levels of several other miRNAs which are not included in these clusters (Additional file 8d). These data suggest that the change of miRNA expression under the chronic mitophagic flux is not a general nonspecific phenomenon affecting all miRNAs. Taken together, miR-27a/b are endogenous inhibitors of mitophagy and may function as a negative feedback loop under chronic mitophagic flux (Fig. 9c).

## Discussion

Mitochondria play critical roles in a variety of cellular functions, such as energy production, calcium homeostasis, and apoptosis [44]. Mitochondrial dysfunction has been suggested as a key factor in several diseases, including metabolic diseases and neurodegenerative diseases [44, 45]. Therefore, maintaining mitochondrial homeostasis is critical for cellular functions and health. In eukaryotic cells, mitochondrial homeostasis is maintained by mitochondrial quality control system which is a dynamic process coordinated by fission and fusion, mitophagy, transport, and biogenesis [46].

PINK1 plays critical roles in mitochondrial quality control, particularly in induction of mitophagy [39]. Although post-translational stabilization of PINK1 upon mitochondrial damage has been extensively studied, our understanding of PINK1 expression regulation at the transcriptional or translational levels, in particular, by miRNAs is still very limited. Here, we demonstrated that miR-27a/b negatively regulate human PINK1 through translational inhibition by directly targeting 3′UTR of its mRNA. Of note, our data suggest that PINK1 translation is critical for the accumulation of PINK1 protein upon mitochondrial damage. We further demonstrated that translational inhibition of PINK1 by miR-27a/b suppresses the autophagic clearance of damaged mitochondria by lysosome.

Our results demonstrated that endogenous PINK1 levels are actively regulated by the expression levels of miR-27a/b under the basal condition. Inhibition of endogenous miR-27a/b significantly increased the PINK1 protein levels under the basal condition. These data suggest that endogenous miR-27a/b critically contribute to maintaining the level of PINK1 protein lower than the threshold required for mitophagic induction and thereby, may protect cells from the untoward loss of mitochondria under the basal condition. Upon mitochondrial damage, mitophagic influx is regulated by miR-27a/b through their direct regulation of PINK1 expression, suggesting the roles of miR-27a/b as gate-keepers of mitophagic pathway. Interestingly, the expression of miR-27a/b is dramatically increased under the chronic mitophagic flux condition. Given the critical roles of mitochondria in cellular homeostasis and energy metabolism, the continuous loss of mitochondria will be toxic to the cells. Our data suggest that miR-27a/b may function as a negative feedback loop under the chronic mitophagic flux condition in order to protect cells from the depletion of mitochondria. Determining how the expression of miR-27a/b is regulated under the basal condition and mitochondrial stress condition will provide further insights into the regulatory mechanism of PINK1 and mitophagy.

miRNAs are getting more attention as critical regulators for many cellular processes. So far, only few miRNAs have been shown to regulate mitophagy. For example, miR-137 inhibits mitophagy by targeting FUNC1 and NIX [47]. miR-320a promotes mitophagy by targeting VDAC1 [48]. miR-351 and miR-125a are also known to regulate mitophagy [49]. These studies suggest that mitophagy is actively regulated by diverse miRNAs at multiple levels. In this study, we report that endogenous miR-27a/b play a critical role in PINK1 expression, thereby in mitophagy. Therefore, our study significantly contributes to understanding of the regulation mechanism of mitophagy, in particular, by miRNAs. Loss of PINK1 has been associated with mitochondrial dysfunction in *Drosophila*, mouse, and human, suggesting that the roles of PINK1 in mitochondrial quality control may be evolutionally conserved [50, 51]. Although the function of PINK1 may be conserved across evolution, our study suggests that miR-27a/b may exert a primate-specific regulation of PINK1 and mitophagy. miR-27a and miR-27b are produced from miR-23a ~ 27a ~ 24–2 and miR-23b ~ 27b ~ 24–2 cluster, respectively. These paralogous clusters also produce miR-23a, miR-23b, and miR-24. Interestingly, all of them are known to regulate autophagy-associated genes. For example, miR-23a, miR-23b, and miR-24 suppress AMBRA1 [52], ATG12 [53, 54], and ATG4A [55], respectively. Although the role of ATG4A in mitophagy is unclear, AMBRA1 and ATG12 positively regulate mitophagy [56–58]. Therefore, it is tempting to speculate that miRNAs in those clusters may coordinate their function to effectively regulate mitophagy.

PINK1 is expressed ubiquitously in neurons throughout the human brain [59, 60]. Several previous studies showed that miR-27a/b are expressed in human midbrain [34–36]. Because we demonstrated that endogenous miR-27a/b regulate PINK1 expression in human dopaminergic-like M17 cells, our study raises the possibility that miR-27a/b may regulate PINK1 expression in dopaminergic neurons of the human midbrain. Although the role of PINK1 in mitophagy in neurons is controversial [61], PINK1 also plays crucial roles in mitochondria trafficking, spine morphogenesis, and vulnerability to excitotoxicity in neurons [23]. Therefore, further studies are warranted to determine the roles of miR-27a/b in dopaminergic neurons of the human brain.

Recent miRNA profiling studies identified several miRNAs that are dysregulated in the affected regions of PD brains [35, 36, 62]. However, there are significant discrepancies between these studies, likely due to methodological differences as well as the heterogeneity of sample population, disease stages, and brain subregions examined. Moreover, relatively small sample sizes in those studies make it difficult to draw a firm conclusion whether expression of miR-27a/b and other miRNAs in miR-23a ~ 27a ~ 24–2 and miR-23b ~ 27b ~ 24–2 cluster are dysregulated in the brains of PD patients. Closer examination of miR-27a/b expression in different brain cell types at different stages of PD is needed to gain further insight into the putative roles of miR-27a/b in PD pathogenesis.

## Conclusions

PINK1 is genetically associated with Parkinson’s disease (PD) and plays critical roles in maintaining mitochondrial homeostasis. In this study, we identified a novel regulatory mechanism of PINK1 by miR-27a and miR-27b at the translational level. Our research highlights that protein translation step of PINK1 is critical for PINK1 accumulation upon mitochondrial damage. Consequently, the accumulation of PINK1 and mitophagic flux upon mitochondrial damage is strongly regulated by expression levels of miR-27a and miR-27b. Our results represent a significant advance in our understanding of translational control of PINK1 expression and mitophagy by miRNAs.

## Methods

### Cell culture

Human cervical HeLa and dopaminergic-like M17 cells were grown in Dulbecco’s modified Eagle’s medium (DMEM, Invitrogen, 11965084) or Opti-MEM I (Invitrogen, 31985070) with 10 % fetal bovine serum (FBS, Invitrogen, 16000044) and 1 % penicillin/streptomycin at 37 °C in a humidified 5 % CO_2_ incubator, respectively. Synthetic miR-27a/b and a negative control miR (Catalog # M-03-D) were from Insight Genomics. Anti-miR-27a/b (4101393–001) and anti-negative control (199006–001) were from Exiqon. AGO2 (E-004639-00-0005), DICER1 siRNA (M-003483-00-0005), and negative siRNA control (D-001206-14-05) were from Dharmacon. PINK1 siRNA (5′-GACGCTGTTCCTCGTTATGAA-3′) was from Qiagen.

### Quantitative Real Time Polymerase Chain Reaction (qRT-PCR)

Total RNAs were extracted using TRIzol® Reagent (Invitrogen) and reverse transcribed with High Capacity cDNA Reverse Transcription kit (Applied Biosystems) for mRNAs or with Mir-X™ miRNA First-Strand Synthesis Kit (Clontech) for miRNAs except miR-27a/b according to the manufacturer’s guides. Quantitative PCR was performed with Power SYBR Green PCR Master Mix, ABI 7500, and ABI 7900 (Applied Biosystems) using default thermal cycling program. *PINK1* mRNA levels were measured with PINK1 forward primer: GGAGGAGTATCTGATAGGGCAG and reverse primer: AACCCGGTGCTCTTTGTCAC. miR-27a/b levels were measured as previously described [63]. Mature sequences and universal reverse primer were used for all other miRNAs. *GAPDH* and *U6* were used as normalization controls for mRNA and miRNA, respectively.

### AGO-specific RNA immunoprecipitation assay

RNA immunoprecipitation assay was carried out as previously described [64]. Briefly, HeLa cells were transfected with 50 nM of AGO2 siRNA or negative control. 48 h after transfection, AGO2-bound mRNAs were pulled down from cell lysates with 2A8 anti-AGO2 antibody. Then, RNAs were extracted using TRIzol® Reagent and PINK1 mRNA levels were measured by qRT-PCR.

### Luciferase assay

The entire 3′UTR of human *PINK1* mRNA (NM_032409, 1841–2660) was cloned into psiCHECK^TM^-2 vector at XhoI/NotI site downstream of *Renilla* luciferase. psiCHECK^TM^-2 vector also contains a constitutively expressed firefly luciferase gene that was used to normalize *Renilla* luciferase activity. Mutations in seed match sites were introduced using KOD-Plus-Mutagenesis Kit (TOYOBO). HeLa cells were plated at a density of 2.4 × 10^4^ cells per well in a 96-well plate a day before transfection. Cells were transfected with 40 nM of miR-27a/b or negative control or with 150 nM of anti-miR-27a/b or anti-control along with 40 ng of luciferase reporter vector as indicated. 48 h post-transfection, luciferase activity was measured using the Dual-Glo Luciferase Assay System (Promega).

### Mitochondrial fractionation

48 h post-transfection as indicated, cell were treated with 10 μM CCCP for 2 h and then, mitochondrial fraction was prepared as previously described [65].

### High Content Imaging and immunofluorescence staining

For quantification of Parkin translocation, 40 nM miRNA was transfected to HeLa cells stably expressing GFP-Parkin in 384-well imaging plates. 48 h post-transfection, cells were treated with 10 μM CCCP for 2 h. Cells were then fixed in 4 % paraformaldehyde, stained with Hoechst 33342 (Invitrogen), and imaged on a BD Pathway 855 system (Becton Dickinson Biosciences). Parkin translocation was analyzed as previously described [40]. Analyzed were 4 independently transfected wells each with a total number of > 1000 cells per condition. After imaging on the BD pathway, plates were stained with mitochondrial marker, translocase of outer mitochondrial membrane 20 homolog (yeast) (Tom20), using anti-TOM20 antibody (Proteintech, 11802-1-AP). Confocal fluorescent images were taken with a 40 x Plan-Apochromat objective using a Zeiss AxioObserver equipped with an ApoTome Imaging System (Zeiss).

For the analysis of mitochondrial degradation, delivery of the damaged mitochondria to lysosome was visualized and quantified as previously described [41] with some modifications. Briefly, 40nM miRNA was transfected in HeLa cells stably expressing mitochondria-targeting Keima (mtKeima) in 384-well imaging plates. 48 h post-transfection, cells were treated with 4 μM CCCP for 12 h. Cells were imaged live after addition of Hoechst 33342 on the BD Pathway system. Acquisition was performed with a 2x2 montage (no gaps) after laser autofocus. 440/10 nm and 548/20 nm excitation filter were used for neutral and acidic Keima, respectively. Emission was filtered through a 595 nm longpass dichroic filter. 570 nm and 645/75 nm emission filters were used for neutral and acidic Keima, respectively. Raw images were processed using the build-in AttoVision V1.6 software. Regions of interest (ROIs) were defined as nucleus and cytoplasm using the build-in ‘RING - 2 outputs’ segmentation for the Hoechst channel after applying a shading algorithm. The signal intensity of the acidic Keima in the cytoplasm was divided by the intensity of the neutral mtKeima. Analyzed were 12 wells per condition with a minimum of 300 cells per well. PINK1 siRNA was used as a positive control for both assays.

### Western blot analysis

Western blots were performed as previously described [66]. All membranes were blocked with 4 % non-fat dry milk in TBS/T (Tris buffered saline with 0.125 % Tween-20) except for pUb^S65^ which was blocked with 4 % bovine serum albumin (Jackson ImmunoResearch, 001-000-173) in TBS/T. Blots were probed in blocking buffer with anti-Dicer (Cell Signaling Technology, 5362), anti-PINK1 (Novus Biologicals, BC100-494), anti-SOD2 (Abcam, ab13533), anti-VDAC1 (Abcam, ab14734), anti-p38 (Cell Signaling Technology, 9212), anti-COX2 (Abcam, ab110258), or anti-GAPDH antibody (Santa Cruz, sc-25778). pUb^S65^-specific antibody were produced and characterized as previously described [19].

### Statistical analysis

Statistical analyses were performed using two-tailed Student’s *t*-test for the comparison of two groups. The comparison of multiple groups more than two were analyzed using one-way ANOVA or two-way ANOVA test depending on comparison variables with Tukey’s pot-hoc analysis as indicated (GraphPad Prism 5).

## Abbreviations

AGO, Argonaute; CCCP, carbonyl cyanide m-chlorophenylhydrazone; COX2, cytochrome c oxidase 2; Dicer, dicer 1, ribonuclease type III; GAPDH, glyceraldehyde-3-phosphate dehydrogenase; GFP, green fluorescent protein; LRRK2, Leucine-rich repeat kinase 2; miR-27a, microRNA-27a; miR-27b, microRNA-27b; miRNA, microRNA; ORF, open reading frame; PAGE, polyacrylamide gel electrophoresis; PD, Parkinson’s disease; PINK1, PTEN-induced putative kinase 1; qRT-PCR, quantitative Real Time Polymerase Chain Reaction; SNCA, synuclein alpha; TOM20, translocase of outer mitochondrial membrane 20 homolog (yeast); UTR, untranslated region; VDAC1, voltage-dependent anion channel 1

## Additional files

Additional file 1: Knock-down of *DICER1* decreased miRNA levels. HeLa cells were transfected with 50 nM of *DICER1* siRNA (DICER1 KD) or negative control (Ctl). 48 h post-transfection, representative miRNA levels were analyzed by qRT-PCR and were quantified as a percentage of control (*n* = 2). Values are mean ± SEM. (PDF 18 kb) Additional file 2: Computational prediction of miRNA candidates for human PINK1. **a** Computation prediction of miRNAs expressed in human midbrain with putative binding sites in the 3′UTR of human *PINK1* mRNA. We first searched miRNAs that have putative binding sites in the 3′UTR of human *PINK1* mRNA by utilizing several miRNA-target prediction algorithms, such as miRanda [67], miRWalk [68], RNAhybrid [37], and Targetscan [69]. Among 49 miRNAs commonly predicted by different algorithms, 7 miRNAs were known to be expressed in human midbrain [34]. miR-27a/b are predicted to have 2 putative binding sites in the 3′UTR of human *PINK1* mRNA, while all other miRNAs are predicted to have 1 putative binding site. **b** Computational binding prediction of miR-27a/b and their binding sites in the 3′UTR of human *PINK1* mRNA. The binding free energies were determined by the RNAhybrid algorithm. (PDF 68 kb) Additional file 3:
**a** Comparison of 3′UTR sequences of *PINK1* mRNAs from different species. Multiple alignment was performed using ClustalW program with 3′UTR sequences of *PINK1* mRNAs from human (NM_032409), chimpanzee (XM_001164912), monkey (XM_001096957), rat (NM_001106694), and Mouse (NM_026880). Seed match sites for miR-27a/b are highlighted in red. **b** A phylogenetic tree of 3′UTR sequences of *PINK1* mRNA from different species. The neighbor-jointing tree for the full-length 3′UTR sequences of *PINK1* mRNAs was generated with ClustalW program. (PDF 27 kb) Additional file 4: The levels of miR-27a (**a**) and miR-27b (**b**) in the luciferase assay. HeLa cells were transfected with miR-27a/b or negative control (Ctl) along with the reporter constructs as indicated in the X axis. 48 h post-transfection, miR-27a/b levels were measured by qRT-PCR. Each level was normalized to the corresponding *U6* level. Data are shown as a fold change relative to the control miR (*n* = 5, one-way ANOVA). Values are mean ± SEM (n.s. = non-significant). (PDF 29 kb) Additional file 5: The levels of miR-27a/b after overexpression (**a**) and inhibition (**b**) of miR-27a/b. HeLa cells were transfected with 40 nM of negative control (Ctl) or miR-27a/b (A) or with 150 nM of anti-control (anti-Ctl) or anti-miR-27a/b (B). 48 h post-transfection, miR-27a/b levels were determined by qRT-PCR. Each level was normalized to the corresponding *U6* level. Data are shown as a fold change relative to control (*n* = 4). Values are mean ± SEM (n.s. = non-significant, ****p* < 0.001, one-way ANOVA (A) or *t*-test (B)). (PDF 28 kb) Additional file 6: A combination of miR-27a and miR-27b does not further inhibit PINK1 expression compared to each miRNA alone. HeLa cells were transfected with 80 nM negative control (Ctl), 40 nM Ctl with 40 nM miR-27a (miR-27a), 40 nM Ctl with 40 nM miR-27b (miR-27b), or 40 nM miR-27a with 40 nM miR-27b (miR-27a/b). 48 h post-transfection, PINK1 levels were measured by Western blot and normalized to corresponding GAPDH levels. Data are shown as a percentage of control (*n* = 4, one-way ANOVA). Values are mean ± SEM (****p* < 0.001). (PDF 42 kb) Additional file 7: Knock-down of PINK1 prevents the delivery of damaged mitochondria to lysosome. 48 h post-transfection with negative control (si-Ctl) or PINK1 siRNA, HeLa cells stably expressing mtKeima were treated with 4 μM CCCP or DMSO for 12 h. Cells were sequentially scanned using 440/10 nm (neutral) and 548/20 nm (acidic) excitation filters. Scale bars correspond to 100 μm. The signal intensity of the acidic Keima was divided by the intensity of the neutral mtKeima (**a**). Data were collected from 12 independent replicates and are shown as a percentage of control (n > 300 cells, two-way ANOVA) (**b**). Values are mean ± SEM (n.s. = non-significant, ****p* < 0.001). (PDF 291 kb) Additional file 8:
**a** Schematic diagram of miR-23a ~ 27a ~ 24–2 and miR-23b ~ 27b ~ 24–1 clusters. **b**, **c** The levels of miR-23a/b upon mitochondrial damages. HeLa cells were incubated with DMSO, 10 μM CCCP, or combination of 10 μM oligomycin and 4 μM antimycin as indicated. miR-23a/b levels were measured by qRT-PCR and normalized to corresponding *U6* levels. Data are shown as a fold change relative to DMSO control. **d** The levels of miR-103, miR-221, and miR-7a upon mitochondrial damages were measured by qRT-PCR and normalized to corresponding *U6* levels. Values are mean ± SEM (*t*-test, ****p* < 0.001). (PDF 41 kb)
