# Supplementary figures and images for: miR-27a and miR-27b regulate autophagic clearance of damaged mitochondria by targeting PTEN-induced putative kinase 1 (PINK1)
Source: Mol Neurodegener. 2016 Jul 26;11:55. doi: 10.1186/s13024-016-0121-4 (PMC4960690; doi:10.1186/s13024-016-0121-4)

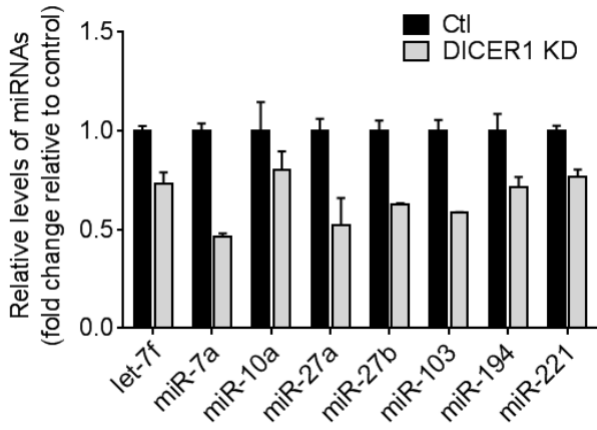

Supplement: Additional file 1: — Knock-down of DICER1 decreased miRNA levels. HeLa cells were transfected with 50 nM of DICER1 siRNA (DICER1 KD) or negative control (Ctl). 48 h post-transfection, representative miRNA levels were analyzed by qRT-PCR and were quantified as a percentage of control (n = 2). Values are mean ± SEM. (PDF 18 kb) [file 13024_2016_121_MOESM1_ESM.pdf]

**A**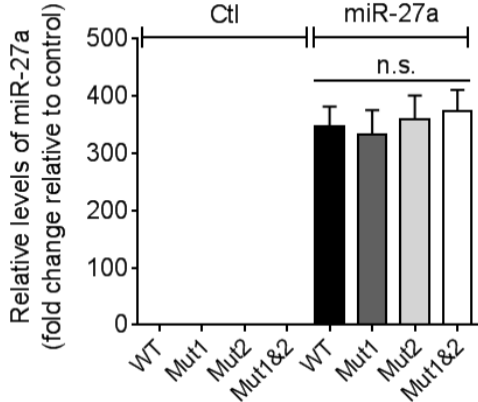**B**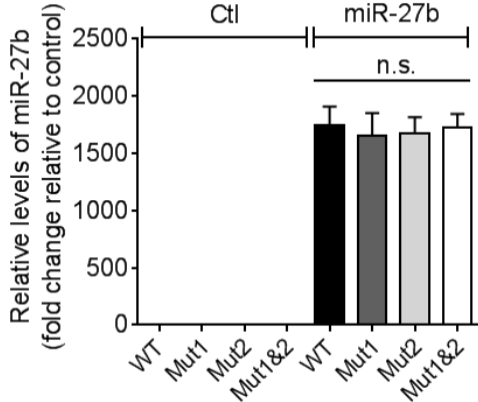

Supplement: Additional file 4: — The levels of miR-27a (a) and miR-27b (b) in the luciferase assay. HeLa cells were transfected with miR-27a/b or negative control (Ctl) along with the reporter constructs as indicated in the X axis. 48 h post-transfection, miR-27a/b levels were measured by qRT-PCR. Each level was normalized to the corresponding U6 level. Data are shown as a fold change relative to the control miR (n = 5, one-way ANOVA). Values are mean ± SEM (n.s. = non-significant). (PDF 29 kb) [file 13024_2016_121_MOESM4_ESM.pdf]

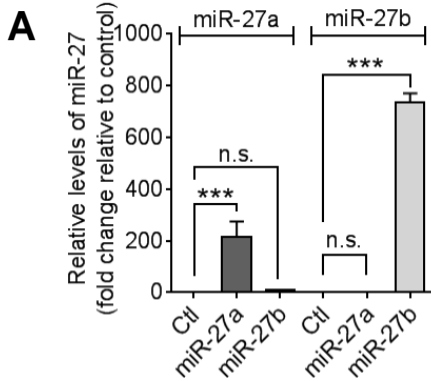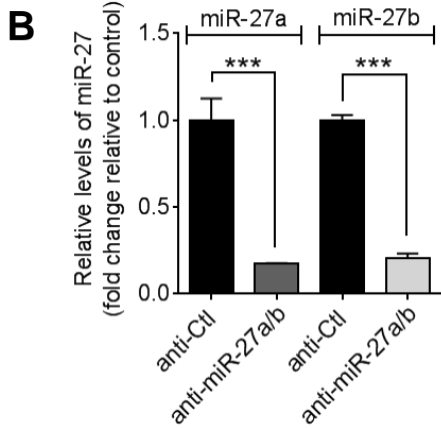

Supplement: Additional file 5: — The levels of miR-27a/b after overexpression (a) and inhibition (b) of miR-27a/b. HeLa cells were transfected with 40 nM of negative control (Ctl) or miR-27a/b (A) or with 150 nM of anti-control (anti-Ctl) or anti-miR-27a/b (B). 48 h post-transfection, miR-27a/b levels were determined by qRT-PCR. Each level was normalized to the corresponding U6 level. Data are shown as a fold change relative to control (n = 4). Values are mean ± SEM (n.s. = non-significant, ***p < 0.001, one-way ANOVA (A) or t-test (B)). (PDF 28 kb) [file 13024_2016_121_MOESM5_ESM.pdf]

**A**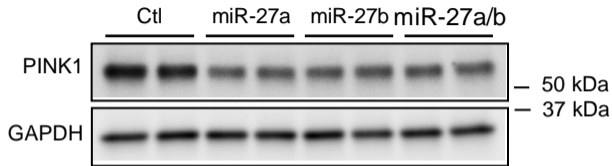**B**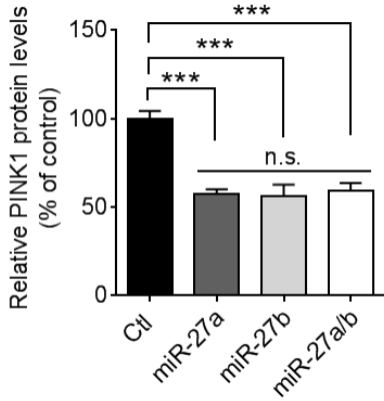

Supplement: Additional file 6: — A combination of miR-27a and miR-27b does not further inhibit PINK1 expression compared to each miRNA alone. HeLa cells were transfected with 80 nM negative control (Ctl), 40 nM Ctl with 40 nM miR-27a (miR-27a), 40 nM Ctl with 40 nM miR-27b (miR-27b), or 40 nM miR-27a with 40 nM miR-27b (miR-27a/b). 48 h post-transfection, PINK1 levels were measured by Western blot and normalized to corresponding GAPDH levels. Data are shown as a percentage of control (n = 4, one-way ANOVA). Values are mean ± SEM (***p < 0.001). (PDF 42 kb) [file 13024_2016_121_MOESM6_ESM.pdf]

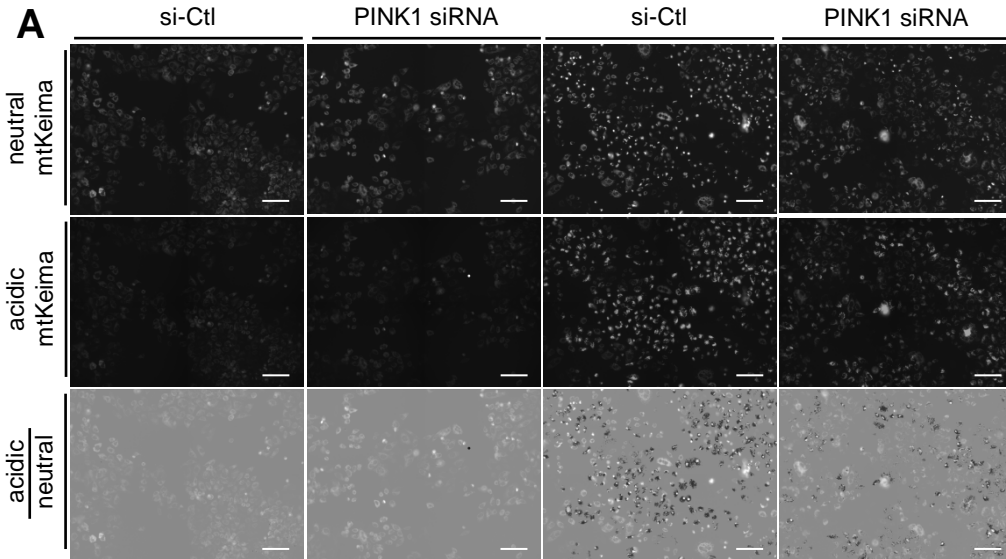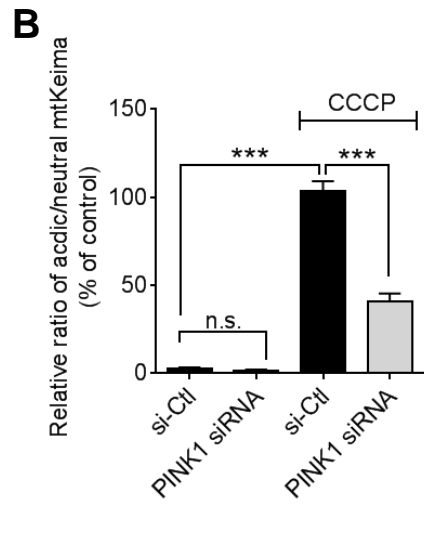

Supplement: Additional file 7: — Knock-down of PINK1 prevents the delivery of damaged mitochondria to lysosome. 48 h post-transfection with negative control (si-Ctl) or PINK1 siRNA, HeLa cells stably expressing mtKeima were treated with 4 μM CCCP or DMSO for 12 h. Cells were sequentially scanned using 440/10 nm (neutral) and 548/20 nm (acidic) excitation filters. Scale bars correspond to 100 μm. The signal intensity of the acidic Keima was divided by the intensity of the neutral mtKeima (a). Data were collected from 12 independent replicates and are shown as a percentage of control (n > 300 cells, two-way ANOVA) (b). Values are mean ± SEM (n.s. = non-significant, ***p < 0.001). (PDF 291 kb) [file 13024_2016_121_MOESM7_ESM.pdf]

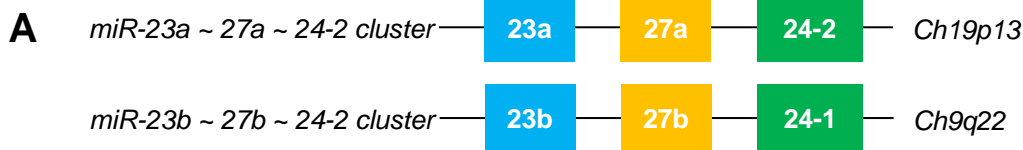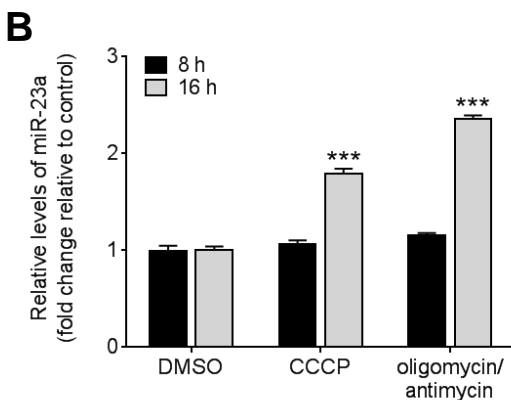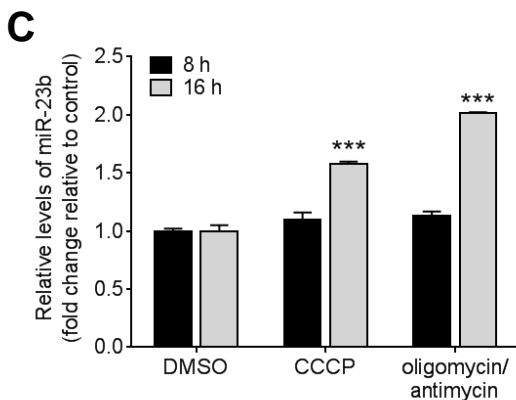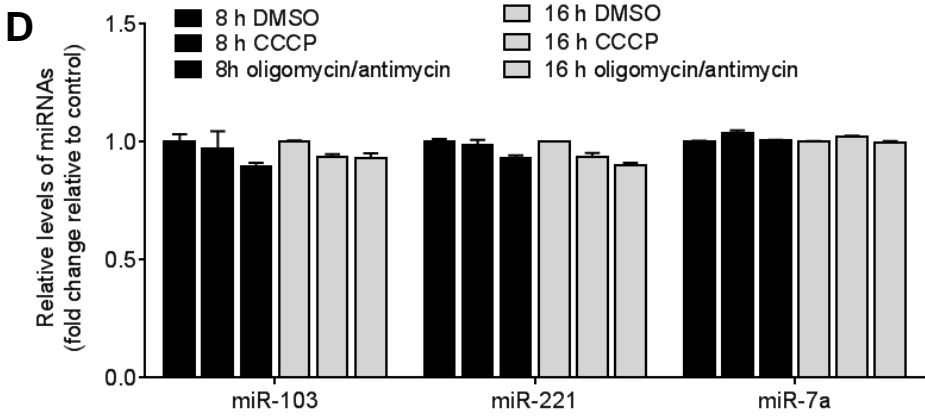

Supplement: Additional file 8: — a Schematic diagram of miR-23a ~ 27a ~ 24–2 and miR-23b ~ 27b ~ 24–1 clusters. b, c The levels of miR-23a/b upon mitochondrial damages. HeLa cells were incubated with DMSO, 10 μM CCCP, or combination of 10 μM oligomycin and 4 μM antimycin as indicated. miR-23a/b levels were measured by qRT-PCR and normalized to corresponding U6 levels. Data are shown as a fold change relative to DMSO control. d The levels of miR-103, miR-221, and miR-7a upon mitochondrial damages were measured by qRT-PCR and normalized to corresponding U6 levels. Values are mean ± SEM (t-test, ***p < 0.001). (PDF 41 kb) [file 13024_2016_121_MOESM8_ESM.pdf]
