# Supplementary material for: miR-27a and miR-27b regulate autophagic clearance of damaged mitochondria by targeting PTEN-induced putative kinase 1 (PINK1)
Source: Mol Neurodegener. 2016 Jul 26;11:55. doi: 10.1186/s13024-016-0121-4 (PMC4960690; doi:10.1186/s13024-016-0121-4)

A

Gene	MicroRNA	miRanda	miRWalk	RNAhybrid	Targetscan	Total prediction
PINK1	hsa-miR-516a-3p	Y	Y	Y	Y	4
PINK1	hsa-miR-346	Y	Y	Y	Y	4
PINK1	hsa-miR-548c-3p	Y	Y	Y	Y	4
PINK1	hsa-miR-124	Y	Y	Y	Y	4
PINK1	hsa-miR-1229	Y	Y	Y	Y	4
PINK1	hsa-miR-340	Y	Y	Y	Y	4
PINK1	hsa-miR-506	Y	Y	Y	Y	4
PINK1	hsa-miR-217	Y	Y	Y	Y	4
PINK1	hsa-miR-324-3p	Y	Y	Y	Y	4
PINK1	hsa-miR-486-5p	Y	Y	Y	Y	4
PINK1	hsa-miR-1205	Y	Y	Y	Y	4
PINK1	hsa-miR-532-3p	Y	Y	Y	Y	4
PINK1	hsa-miR-197	Y	Y	Y	Y	4
PINK1	hsa-miR-632	Y	Y	Y	Y	4
PINK1	hsa-miR-889	Y	Y	Y	Y	4
PINK1	hsa-miR-188-3p	Y	Y	Y	Y	4
PINK1	hsa-miR-1254	Y	Y	Y	Y	4
PINK1	hsa-miR-345	Y	Y	Y	Y	4
PINK1	hsa-miR-519e	Y	Y	Y	Y	4
PINK1	hsa-miR-455-3p	Y	Y	Y	Y	4
PINK1	hsa-miR-598	Y	Y	Y	Y	4
PINK1	hsa-miR-198	Y	Y	Y	Y	4
PINK1	hsa-miR-27b	Y	Y	Y	Y	4
PINK1	hsa-miR-216b	Y	Y	Y	Y	4
PINK1	hsa-miR-509-3-5p	Y	Y	Y	Y	4
PINK1	hsa-miR-1260	Y	Y	Y	Y	4
PINK1	hsa-miR-515-3p	Y	Y	Y	Y	4
PINK1	hsa-miR-605	Y	Y	Y	Y	4
PINK1	hsa-miR-654-3p	Y	Y	Y	Y	4
PINK1	hsa-miR-1180	Y	Y	Y	Y	4
PINK1	hsa-miR-1292	Y	Y	Y	Y	4
PINK1	hsa-miR-431	Y	Y	Y	Y	4
PINK1	hsa-miR-568	Y	Y	Y	Y	4
PINK1	hsa-miR-27a	Y	Y	Y	Y	4
PINK1	hsa-miR-767-3p	Y	Y	Y	Y	4
PINK1	hsa-miR-380	Y	Y	Y	Y	4
PINK1	hsa-miR-1280	Y	Y	Y	Y	4
PINK1	hsa-miR-513a-5p	Y	Y	Y	Y	4
PINK1	hsa-miR-575	Y	Y	Y	Y	4
PINK1	hsa-miR-1236	Y	Y	Y	Y	4
PINK1	hsa-miR-513b	Y	Y	Y	Y	4
PINK1	hsa-miR-409-3p	Y	Y	Y	Y	4
PINK1	hsa-miR-513a-3p	Y	Y	Y	Y	4
PINK1	hsa-miR-588	Y	Y	Y	Y	4
PINK1	hsa-miR-626	Y	Y	Y	Y	4
PINK1	hsa-miR-205	Y	Y	Y	Y	4
PINK1	hsa-miR-142-5p	Y	Y	Y	Y	4
PINK1	hsa-miR-1238	Y	Y	Y	Y	4
PINK1	hsa-miR-1825	Y	Y	Y	Y	4
PINK1	hsa-miR-628-5p	Y	Y	Y	Y	4
PINK1	hsa-miR-509-5p	Y	Y	Y	Y	4
PINK1	hsa-miR-1200	Y	Y	Y	N	3
PINK1	hsa-miR-516a-3p	Y	Y	N	Y	3

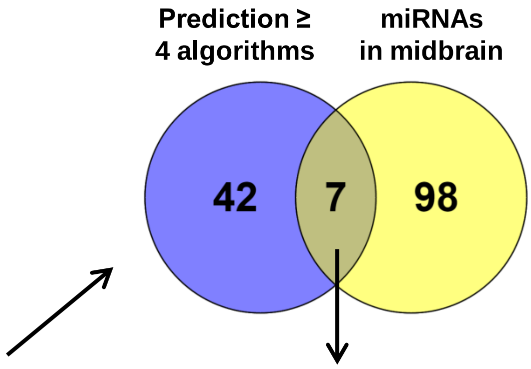

MicroRNA	Match number
hsa-miR-27a	2
hsa-miR-27b	2
hsa-miR-124	1
hsa-miR-197	1
hsa-miR-340	1
hsa-miR-345	1
hsa-miR-486	1

B

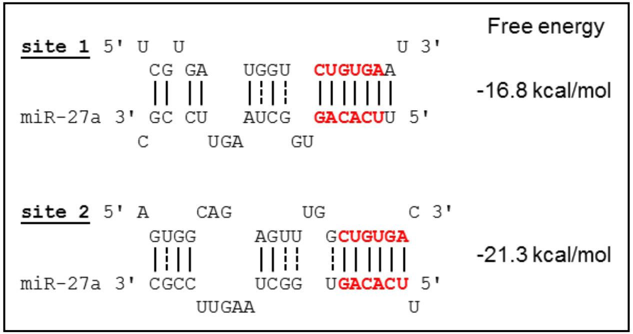

Supplement: Additional file 2: — Computational prediction of miRNA candidates for human PINK1. a Computation prediction of miRNAs expressed in human midbrain with putative binding sites in the 3′UTR of human PINK1 mRNA. We first searched miRNAs that have putative binding sites in the 3′UTR of human PINK1 mRNA by utilizing several miRNA-target prediction algorithms, such as miRanda [67], miRWalk [68], RNAhybrid [37], and Targetscan [69]. Among 49 miRNAs commonly predicted by different algorithms, 7 miRNAs were known to be expressed in human midbrain [34]. miR-27a/b are predicted to have 2 putative binding sites in the 3′UTR of human PINK1 mRNA, while all other miRNAs are predicted to have 1 putative binding site. b Computational binding prediction of miR-27a/b and their binding sites in the 3′UTR of human PINK1 mRNA. The binding free energies were determined by the RNAhybrid algorithm. (PDF 68 kb) [file 13024_2016_121_MOESM2_ESM.pdf]
