# Supplementary material for: miR-27a and miR-27b regulate autophagic clearance of damaged mitochondria by targeting PTEN-induced putative kinase 1 (PINK1)
Source: Mol Neurodegener. 2016 Jul 26;11:55. doi: 10.1186/s13024-016-0121-4 (PMC4960690; doi:10.1186/s13024-016-0121-4)

A

```
Human       --------------------TGTCCCTGCATGGAGCTGGTGAATTACTAAAAGAACATGG 40
Chimpanzee  --------------------TGTCCCTGCATGGAGCTGGTGAATTACTAAAAGAACTTGG 40
Monkey      --------------------TGTCCCTGCATGGAGCTGGTGAATTACTAAAAGAACTTGG 40
Rat         ------------------------------------------------------------
Mouse       TGGCTCTGTGCTCCAGTTACTAAAAGACTAAGGGGCATCTGTGTCCTGATGTCATGACAG 60

Human       CATCCTCTGTGTCGTGATGGTCTGTGAATGGTGAGGGTGGGAGTCAGGAGACAAGACAGC 100
Chimpanzee  CATCCTCTGTGTCGTGATGGTCTGTGAATGGTGAGGGTGGGAGTCAGGAGACAAGACAGC 100
Monkey      CATCCGCTGTGTCGTGATGGTCTGTGAATGGTGAGGGTGGGAGTCAGGAGACAAGATGGC 100
Rat         ------------------------------------------------------------
Mouse       TATACGAAGGCTGAAGAAGGTGGCTTGGCTGGGTGGGCAGGAGTCAGGGGACAAGCTGGA 120

Human       GCAGAGAGGGCTGGTTAGCCGGAAAAGGCCTCGGGCTTGGCAAATGGAAGAACTTGAGTG 160
Chimpanzee  GCAGAGAGGGCTGGTTAGCCGGAAAAGGCCTCGGGCTTGGCAAATGGAAGAACTTGAGTG 160
Monkey      GCAGAGAGGGCTGGTTAGCCAGAAAAAGCTTCAGGCTTGGCAAATGGAAGAACTTGAGTC 160
Rat         ------------------------------------------------------------
Mouse       GTAGAGGGAGG-GGCTGGTCAGCCTTGGGCTCAGCAAATGGGAAGAACAGCCTTGAACAG 179

Human       AGAGTTCAGTCTGCAGTCCTCTGCTCACAGACATCTGAAAAGTGAATGGCCAAGCTGGTC 220
Chimpanzee  AGAGTTCAGTCTGCAGTCCTGTGCTCACAGACATCTGAAAAGTGACTGGCCAAGCTGGTC 220
Monkey      AGAGTTCAGTCTGCAGTCCTGTGCTCACAGACATCTGAAAAGTGAATGGCCAAGCTGGTC 220
Rat         ------------ACAGTCAC-TACCTATGCCCATCC---ATCTAAGTTCTAGGGCAGAAC 44
Mouse       CTCAGGAGGGAGAGAGGCAC-TACCTGTGCCCGTCC---AGTTAGGTTCTTGGGCAGAAT 235
                          ** *   * *       * **    *  *   *      ** *

Human       TAGTAGATGAGGCTGGACTGAGGAGGGGTAGGCCTGCAT----CCACAGAGAGGATCCAG 276
Chimpanzee  TAGTAGATGAGGCTGGACTGAGGAGGGGTAGGCCTGCAT----CCACATAGAGGATCCAG 276
Monkey      TAGTAGATGAAGCCGAACTGAGGAGGGAGAGGCCTGCAT----CCACATAGAGGCTCCAG 276
Rat         TCAGACGTCAGGCCAGACCAGGAGTCAGTAAAC-TGTA----------CAGGAAATTAAG 93
Mouse       TAAGACATCAGGCCAGACCAGGAGTTGGTAAGC-TATAGAGGGCCAGACAGGGAATGAAG 294
            *   *  * * **   **   *       *  * *  *           **    *  **

Human       GC----CAAGGCACTGGCTGTCA--GTGGCAGAGTTT-GGCTGTGACCTT--TGCCCCTA 327
Chimpanzee  GC----CAAGGCACTGGCTGTCA--GTGGCAGAGTTT-GGCTGTGACCTT--TGCCCCTA 327
Monkey      GC----CAAGGCACTGGCTGTCA--GTGGCAGAGTTT-GGCTGTGACCTT--TGCCCCTA 327
Rat         TAGTTGTGATGACTTAGCTGTCACCGAGGCAGAAG---GACAAACAC--------GGATG 142
Mouse       TAGTTGTAATGACTAACCTATCACTGAGGCAAAGGCCAAGCAAGCACTGCAGTGAGGCCA 354
                    * *      ** ***  * **** *       *    **

Human       ACACGAGGAACTCGTTTGAAGGGGGCA-GCGTAGCATGTCTG-ATTTGCCACCTGGATGA 385
Chimpanzee  ACACGAGGAACTCGTTTGAAGGGGGCA-GCGTAGCATGTCTG-ATTTGCCACCTGGATGA 385
Monkey      ACATGAGGAACTCGTTTGAAGGAGGCA-GCGTAGCATGTCTGCATTTGCCACCTCGATGA 386
Rat         ACACT-GAACTTTACTTGTCAC---TATACTCTTCTCATTTTTCCCGACCACTTAGAAAT 198
Mouse       ACACT-GAACTTTGCTTGTCGTCAATAAGTTCTGCTCATTTTTCCCAACCACTTAGAAAT 413
            ***   * *  *   ***        *       *   * *       **** * **

Human       AGGCAGACATCAACATGGGTCAGCACG----------TTCAGTTACGGGAGTGGGAAAT- 434
Chimpanzee  AGGCAGACATCAACATGGGTCAGCACG----------TTCAGTTACGGGAGTGGGAAAT- 434
Monkey      AGGCAGCTATCAGCATGGGTCAGCACGAGACAGCACATTCAGTTACAGGAGTGGGAAACG 446
Rat         GCAAAATAACCAGGTTTAGTTTGTAAGTGGC--------CAGTGTGGCTCACAGCCAAG- 249
Mouse       GCAAAAACAT-AGGTTTAATTTGTAAGCAGCA-----ACCAGCATGGCTCATAGTCAAA- 466
                *   *  *   *   *  * * *            ***           *  **

Human       --TACATGAGGCCTGGGCCTC-TGCGTTCCCAAGCTGTGCGTTCTGGACCAGCTACTGAA 491
Chimpanzee  --TACATGAGGCCTGGGCCTC-TGCGTTCCCAAGCTGTGCGTTCTGGACCAGCTACTGAA 491
Monkey      TCTGTGTGAGGCCTGGGCCTC-TGTATTCCCAAGCTGTGTGTTCTGGACCAGCTACTGAA 505
Rat         -TTTGCCCCATCCTGGGCTAAATGCATTCTCAATGTCTAACGCTTAAA--AGATGCTATC 306
Mouse       -TTTACCAATTCCTGGGCTAAATGCATTCTTTATGTCTAACTCTTTAA--AGATGCGAGC 523
              *        *******    **  ***   *  * *      *  *  ** * *

Human       TTATTAATC---TCACTTAGCGAAAGTGACGGATGAGCAGTAAGTAAGTAAGTGTGGGGA 548
Chimpanzee  TTATTAATCATCTCACTTAGCGAAAGTGACTGATGAGCAGTAAGTA----AGTGTGGGGA 547
Monkey      T---TAATC---TCACATAGCGAAAGTGACTGATGAGCAGTAAGT--------GTGGGGA 551
Rat         ATTAAAGACAG-TCTCCAAGCCAAAGCCATG----------------------------- 336
Mouse       ATTAAAGACAG-TCTCCATGCCAAA----------------------------------- 547
                 *  *   ** *   ** ***

Human       TTTAAACTTGAGGGTTTCCCTCCTGACTAGCCTCTCTTACAGGAATTGTGAAATATTAAA 608
Chimpanzee  TTTAAACTTGAGGGTTTCCCTCCTGACTAGCCTCTCTTACAGGAATTGTGAAATATTAAA 607
Monkey      TTTACATTTGAGGGCTTCCCTCCTGACCAGCCTCTCTTACAGGAACTGTGAAATATTAAA 611
Rat         ------------------------------------------------------------
Mouse       ------------------------------------------------------------

Human       TGCAAATTTACAACTGCAGATGACGTATGTGCCTTGAACTGAATATTTGGCTTTAAGAAT 668
Chimpanzee  TGCAAATTTACAACTGCAGA---------------------------------------- 627
Monkey      TGCAAATTTACAACTGCA------------------------------------------ 629
Rat         ------------------------------------------------------------
Mouse       ------------------------------------------------------------

Human       GATTCTTATACTCTGAAGGTGAGAATATTTTGTGGGCAGGTATCAACATTGGGGAAGAGA 728
Chimpanzee  ------------------------------------------------------------
Monkey      ------------------------------------------------------------
Rat         ------------------------------------------------------------
Mouse       ------------------------------------------------------------

Human       TTTCATGTCTAACTAACTAACTTTATACATGATTTTTAGGAAGCTATTGCCTAAATCAGC 788
Chimpanzee  ------------------------------------------------------------
Monkey      ------------------------------------------------------------
Rat         ------------------------------------------------------------
Mouse       ------------------------------------------------------------

Human       GTCAACATGCAGTAAAGGTTGTCTTCAACTGAAAAAAAAAAAAAAAAAAAAA 840
Chimpanzee  ----------------------------------------------------
Monkey      ----------------------------------------------------
Rat         ----------------------------------------------------
Mouse       ----------------------------------------------------
```

B

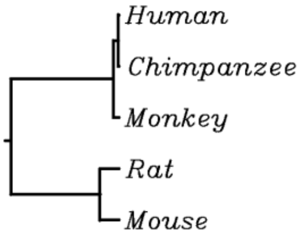

Supplement: Additional file 3: — a Comparison of 3′UTR sequences of PINK1 mRNAs from different species. Multiple alignment was performed using ClustalW program with 3′UTR sequences of PINK1 mRNAs from human (NM_032409), chimpanzee (XM_001164912), monkey (XM_001096957), rat (NM_001106694), and Mouse (NM_026880). Seed match sites for miR-27a/b are highlighted in red. b A phylogenetic tree of 3′UTR sequences of PINK1 mRNA from different species. The neighbor-jointing tree for the full-length 3′UTR sequences of PINK1 mRNAs was generated with ClustalW program. (PDF 27 kb) [file 13024_2016_121_MOESM3_ESM.pdf]
